# Prognostic Value of Ki-67 Index in Patients With Endometrial Stromal Sarcoma

**DOI:** 10.3389/fmed.2021.823505

**Published:** 2022-01-25

**Authors:** Yu Meng, Quan Quan, Fenfen Zhang, Yao Liu, Siling Ren, Xiaoling Mu

**Affiliations:** ^1^Department of Gynecology, The First Affiliated Hospital of Chongqing Medical University, Chongqing, China; ^2^Department of Obstetrics and Gynecology, Chengdu First People Hospital, Chengdu, China; ^3^Department of Obstetrics, Chongqing Fuling District Maternal and Child Health Care, Chongqing, China

**Keywords:** endometrial stromal sarcoma, recurrence, predict, prognosis, Ki-67

## Abstract

**Objective:**

The Ki-67 index is used to evaluate cell proliferation activity, which is related to tumor progression, metastasis, and prognosis. We aimed to explore the prognostic value of Ki-67 index in endometrial stromal sarcoma and to explore the optimal cut-off value of Ki-67 index for predicting recurrent endometrial stromal sarcoma.

**Methods:**

A total of 82 patients with endometrial stromal sarcoma who were treated in our hospital were collected. Clinicopathological data of these patients were retrospectively analyzed. Ki-67 index was detected by the immunohistochemical method. Receiver operating characteristic curve and the Youden index were performed to determine the optimal cut-off value of Ki-67 index for predicting recurrent endometrial stromal sarcoma. The Cox regression was performed to analyze risk factors affecting prognosis of endometrial stromal sarcoma. The Kaplan–Meier method and Log-rank test were performed to analyze the survival of patients.

**Results:**

The optimal cut-off value of Ki-67 index for predicting recurrent endometrial stromal sarcoma was 35%. The results of univariate analysis showed that high Ki-67 index (≥35%) was statistically significantly bound up with shorter progress free survival and overall survival. The results of multivariate analysis showed that Ki-67 index (*P* = 0.001) and ovarian preservation (*P* = 0.040) were independent prognostic factors of progress free survival.

**Conclusions:**

A Ki-67 index cut-off of 35% was optimal for predicting recurrent endometrial stromal sarcoma. Ki-67 index may be a useful prognostic marker in endometrial stromal sarcoma.

## Introduction

Endometrial stromal sarcoma is a rare malignant tumor derived from endometrial stromal cells, accounting for 1% of all primary uterine malignancies and 7–25% of all uterine mesenchymal tumors (1, 2). Endometrial stromal sarcoma mostly occurs in perimenopausal women, but a few cases have been found in young women or adolescents (3, 4). Endometrial sarcomas are classified by the latest WHO classification standard into the following three categories: low-grade endometrial stromal sarcoma, high-grade endometrial stromal sarcoma, and undifferentiated uterine sarcoma (1). Low-grade endometrial stromal sarcoma is a hormone-sensitive tumor with an indolent behavior and favorable prognosis. The characteristic manifestation of the tumor is late recurrence, even in Stage I patients. About one-third of the patients have recurrent disease. Therefore, long-term follow-up is needed (5). High-grade endometrial stromal sarcoma has poorer prognosis with higher recurrence rate compared with low-grade endometrial stromal sarcoma, and undifferentiated uterine sarcoma behaves more aggressively (1, 6). Recurrences occur in 23–59% of patients with endometrial stromal sarcoma, and 15–25% of these patients die of recurrent disease (6). Endometrial stromal sarcoma still lacks consensus on the optimal treatment and risk factors related to poor prognosis, owing to its rarity and histopathological diversity. Although some clinicopathological parameters have been reported, most of them are controversial (7, 8). At present, there are no reliable indicators to predict recurrent endometrial stromal sarcoma after treatment. It is necessary to explore potential predictive and prognostic markers of endometrial stromal sarcoma.

Ki-67 is a proliferation marker protein, which expressed in the cell cycle except the G0 phase. Its expression is specific in the cell cycle and can effectively reflect the state of cell proliferation, so it is often used to evaluate the tumor proliferation activity and biological behavior. And its expression can be observed by immunohistochemistry. The Ki-67 index is a marker used to evaluate cell proliferation activity. It is considered as an indicator of biological aggressiveness and is related to tumor progression, metastasis, and prognosis (9–13). The expression of Ki-67 can be observed in various human malignancies (11, 14–16). It has been found that Ki-67 is one of the prognostic markers of breast cancer. The cut-off values of Ki-67 index were also determined as indicators of post-operative management in breast cancer (9, 17). The clinical value of Ki-67 index has also been reported in gynecological malignancies (10, 15, 18). Some scholars believe that Ki-67 is associated with the prognosis of endometrial stromal sarcoma (5, 19). However, there is no recognized cut-off value of the Ki-67 index in endometrial stromal sarcoma. The purpose of this study was to explore the clinical value of the Ki-67 index as a prognostic marker of endometrial stromal sarcoma and to explore the optimal cut-off value of the Ki-67 index for predicting recurrent endometrial stromal sarcoma.

## Methods

### Study Population

This retrospective study was approved by the relevant institutional review committee, and we obtained the informed consent from all the patients.

Clinicopathological data of 82 patients with endometrial stromal sarcoma who were treated in our hospital from June 2009 to March 2020 were retrospectively analyzed. All the patients underwent surgery and were confirmed by histopathology. The following patients were excluded: (1) The patients who did not undergo surgery; (2) The patients who did not have histopathological results; (3) The patients who did not have complete clinicopathological records.

A follow-up plan was developed according to the risk of recurrence. The patients with low-grade endometrial stromal sarcoma were followed up every 4–6 months in the first 3–5 years, and then one time a year; the patients with high-grade endometrial stromal sarcoma or undifferentiated uterine sarcoma were followed up every 3–4 months in the first 2–3 years, two times a year in the second 2–3 years, and one time a year thereafter.

### Paraffin Section and Immunohistochemistry

All specimens were taken from the post-operative tumor tissues of the patients with endometrial stromal sarcoma, and the specimens were formalin fixed within the specified time and then paraffin embedded. Pathological sections and immunohistochemical staining were performed in the Clinical Pathology Center of our university. CONFIRM™ anti KI-67 (30-9) Rabbit Monoclonal Primary Antibody, CONFIRM anti-Estrogen Receptor (ER) (SP1) Rabbit Monoclonal Primary Antibody, and CONFIRM [TM] anti-Progesterone Receptor (ER) (1E2) Rabbit Monoclonal Primary Antibody were used to recognize Ki-67, estrogen receptor (ER), and progesterone receptor (PR) antigens, respectively. Tissue sections were stained with Ventana NexES staining system (USA). The optimal tumor fixation areas and the high cell density areas were selected from paraffin sections. Two experienced pathologists in gynecological tumors used the blind method to evaluate the paraffin sections independently by Aperio ePathology (Germany) scanner (20 × resolution). The staining intensity and the percentage of positive staining tumor cells were recorded. If the evaluation of the two pathologists is inconsistent, the paraffin block needs to be reevaluated and discussed to reach a consensus.

### Statistical Analysis

Statistical analyses were performed with Statistical Product and Service Solutions (SPSS) 25.0 software. All continuous variables were shown by mean ± SD. The independent sample *t*-test was used to compare the continuous variables, which follow the normal distribution. χ^2^ test or Fisher's exact test was used to compare categorical variables. Receiver operating characteristic curve and the Youden index were performed to determine the optimal cut-off value of the Ki-67 index for predicting recurrent endometrial stromal sarcoma. The Cox regression was performed to analyze risk factors affecting prognosis of endometrial stromal sarcoma. The Kaplan–Meier method and Log-rank test were performed to analyze the survival of the patients. Overall survival (OS) was calculated in months between the date of primary surgery and the date of death or last follow-up, while progress-free survival (PFS) was calculated in months between the date of primary surgery and the date of recurrence or death/last follow-up. *P* < 0.05 indicated that the difference was statistically significant.

## Results

A total of 82 patients with endometrial stromal sarcoma who were treated in our hospital were collected. Four patients without surgery were excluded, and four patients who were unable to perform satisfactory tumor reduction surgery and only took biopsy were excluded. The final analysis includes 74 patients with endometrial stromal sarcoma. Clinicopathological features of these patients are listed in Table 1. Most patients have International Federation of Gynecology and Obstetrics (FIGO) Stage I tumors (46/74, 62.16%). The patients with low-grade endometrial stromal sarcoma account for 67.57% (50/74). The median follow-up time of the patients with endometrial stromal sarcoma was 53 months, ranging from 7 to 132 months. There are 17 patients with recurrent endometrial stromal sarcoma, 11 of them died due to recurrence. According to receiver operating characteristic curve and the Youden index, the optimal cut-off value of the Ki-67 index for predicting recurrent endometrial stromal sarcoma was 35% (Figure 1). The area under the curve was 0.743 [95% CI: 0.574–0.912], and the sensitivity, specificity, and accuracy were 57.1, 91.7, and 74.6%, respectively.

**Table 1:** Clinicopathological features of all patients (*n* = 74).

	***n*** **(%)**
**Age (years)**	
Mean (±SD)	43 (±11)
Median (range)	45 (17–71)
**BMI (kg/m2)**	
Mean (±SD)	23.05 (±3.60)
Median (range)	23.05 (15.55–33.25)
**Menopausal status**	
Pre-menopausal	59 (79.73%)
Post-menopausal	15 (20.27%)
**Histological type**	
LG-ESS	50 (67.57%)
HG-ESS	18 (24.32%)
UUS	5 (6.76%)
Unknown	1 (1.35%)
**Tumor size**	
>7cm	18 (24.32%)
≤7cm	43 (58.11%)
Unknown	13 (17.57%)
**FIGO Stage**	
I	46 (62.16%)
II	14 (18.92%)
III	3 (4.06%)
IV	10^*^ (13.51%)
**Route of surgery**	
Laparotomy	42 (53.85%)
Laparoscopy	26 (33.33%)
Laparotomy + Laparoscopy	9 (11.54%)
Robot-assisted	0 (0%)
Others	1^§^ (1.28%)
**Lymphadenectomy**	
Yes	22 (29.73%)
No	52 (70.27%)
**Ovarian preservation**	
Yes	7 (9.46%)
No	67 (90.54%)
**Adjuvant treatment**	
Yes	53 (71.62%)
No	21 (28.38%)
**CD10**	
Positive	62 (83.78%)
Negative	4 (5.41%)
Unknown	8 (10.81%)
**ER**	
Positive	30 (40.54%)
Negative	10 (13.51%)
Unknown	34 (45.95%)
**PR**	
Positive	31 (41.89%)
Negative	9 (12.16%)
Unknown	34 (45.95%)
**Ki-67 index (%)**	
Mean (±SD)	20 (±15)
Median (range)	20 (0–70)
**Recurrence**	
Yes	17 (22.97%)
No	53^#^ (71.62%)

*SD, standard deviation; BMI, body mass index; LG-ESS, low-grade endometrial stromal sarcoma; HG-ESS, high-grade endometrial stromal sarcoma; UUS, undifferentiated uterine sarcoma; ER, estrogen receptor; PR, progesterone receptor*.

* *The stage of one patient was unknown*.

§ *One patient accepted vulvar mass resection*.

# *Four patients were lost to follow-up*.

**Figure 1:**
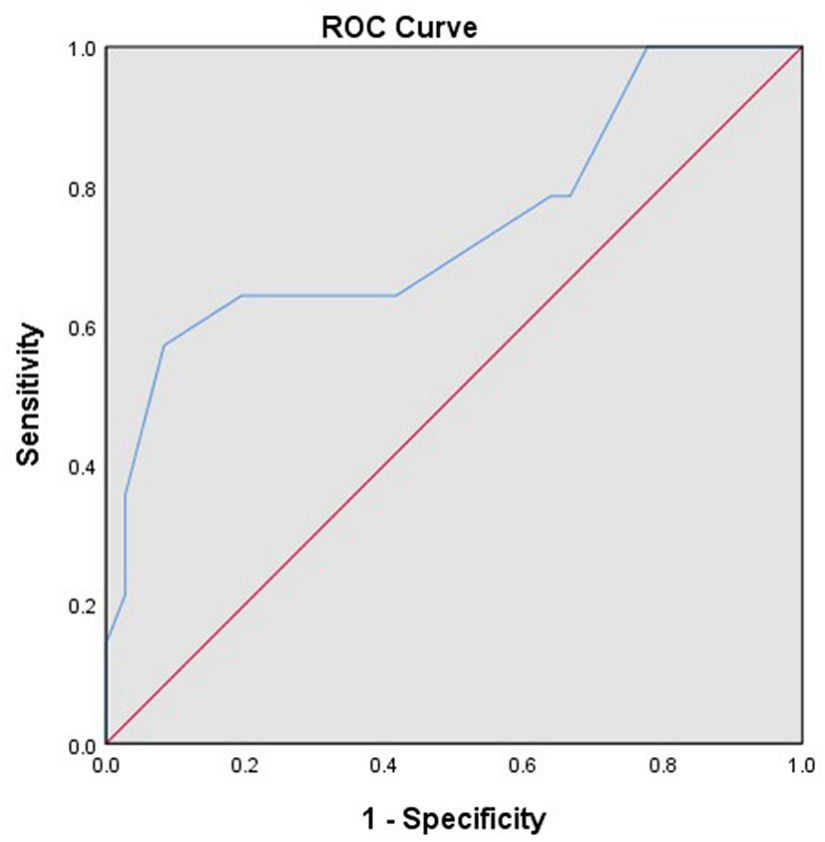
The receiver operating characteristic curve of the Ki-67 index for predicting recurrent endometrial stromal sarcoma.

### Comparison of Clinicopathological Parameters Between the High Ki-67 Index (≥35%) and the Low Ki-67 Index (<35%)

**Figure 2:**
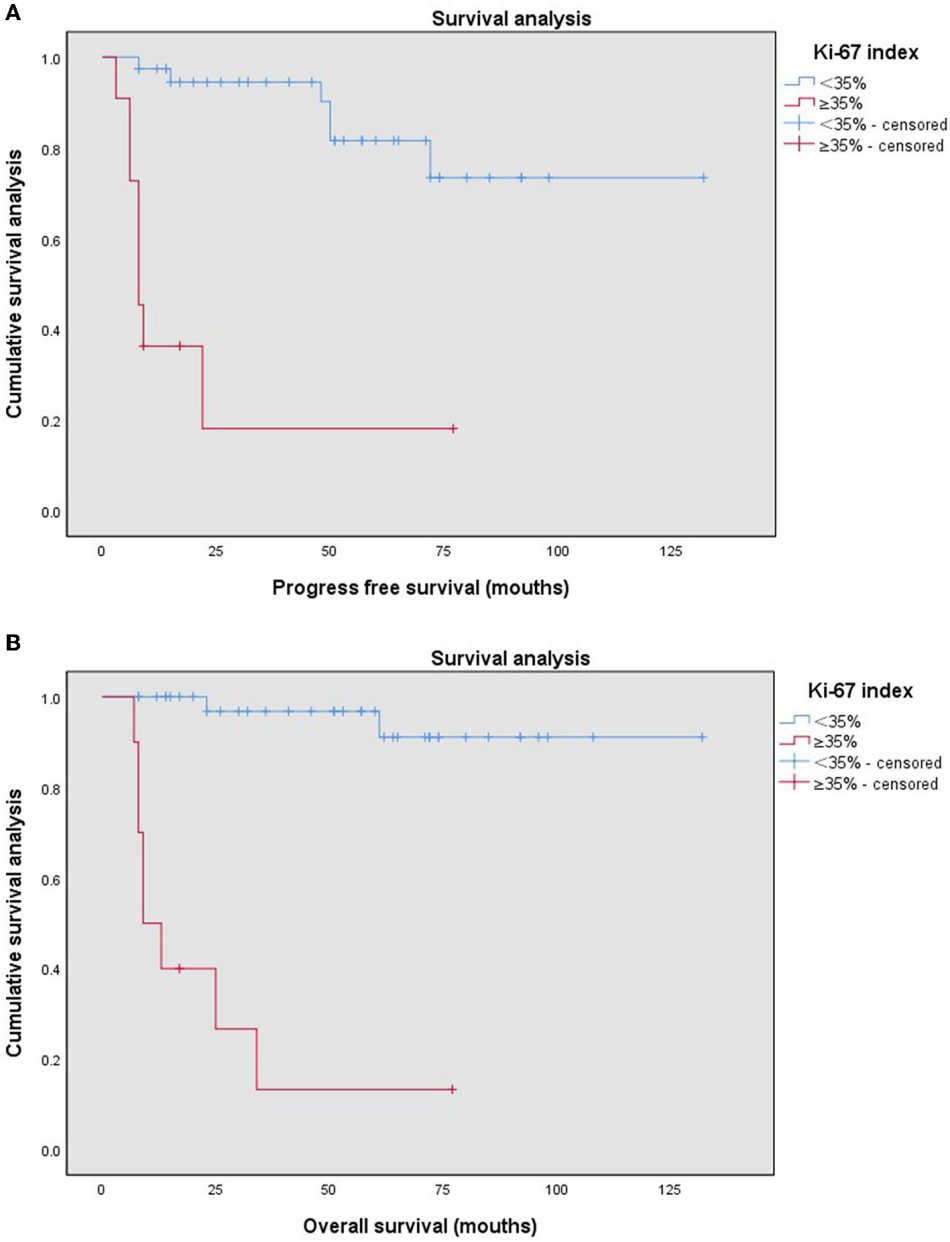
Kaplan–Meier survival curves by different Ki-67 indices (<35%, ≥35%) of the patients with endometrial stromal sarcoma for progression free survival **(A)** and overall survival **(B)**.

According to the cut-off value of the Ki-67 index, the Ki-67 index ≥35% was defined as the high Ki-67 index, and the Ki-67 index <35% was defined as the low Ki-67 index. The high Ki-67 index (≥35%) was significantly associated with the recurrence of endometrial stromal sarcoma (*P* = 0.001). Furthermore, it was also related to body mass index (BMI) (*P* = 0.023), menopausal state (*P* = 0.001), histological type (*P* = 0.000), estrogen receptor (ER) (*P* = 0.000), progesterone receptor (PR) (*P* = 0.000) (Table 2). The median follow-up time of patients with the low Ki-67 index was 57 (8–132) months, while that of the patients with the high Ki-67 index was 11 (7–77) months. Compared with the patients with the low Ki-67 index, the patients with the high Ki-67 index had shorter progress free survival (*P* = 0.000) and overall survival (*P* = 0.000) (Figure 2).

**Table 2:** Relationship between the expression level of the Ki-67 index and clinicopathological characteristics.

**Variables**	**Ki-67 ≥35%**	**Ki-67 < 35%**	***P***
	**(***n*** = 11)**	**(***n*** = 41)**	
Age (years)			0.062
Mean (±SD)	49 (±13)	42 (±10)	
Median (range)	52 (17–63)	44 (20–67)	
BMI (kg/m2)			0.023
Mean (±SD)	25.61 (±3.83)	22.63 (±3.60)	
Median (range)	26.64 (21.33-33.25)	22.63 (15.55–30.36)	
Menopausal status			0.001
Pre-menopausal	4	37	
Post-menopausal	7	4	
Histological type			0.000
LG-ESS	0	35	
HG-ESS	7	5	
UUS	4	1	
Tumor size			0.626
>7cm	4	9	
≤7cm	6	25	
FIGO Stage			0.642
I	7	27	
II	1	10	
III	1	1	
IV	2	3	
Lymphadenectomy			0.118
Yes	6	12	
No	5	29	
Ovarian preservation			1.000
Yes	1	4	
No	10	37	
Adjuvant treatment			0.263
Yes	10	28	
No	1	13	
CD10			0.246
Positive	10	39	
Negative	0	2	
ER			0.000
Positive	1	25	
Negative	7	3	
PR			0.000
Positive	1	26	
Negative	7	2	
Recurrence			0.001
Yes	8	6	
No	3	33	

### Univariate and Multivariate Analyses of Progress-Free Survival

The results of univariate analysis showed that menopausal status (*P* = 0.022), ovarian preservation (*P* = 0.000), histological type (*P* = 0.001), FIGO Stage (*P* = 0.024), estrogen receptor (*P* = 0.002), progesterone receptor (*P* = 0.001), Ki-67 index (*P* = 0.000) were associated with progress-free survival. The results of multivariate analysis showed that ovarian preservation (*P* = 0.040) and the Ki-67 index (*P* = 0.001) were independent prognostic factors of progress-free survival (Table 3).

**Table 3:** The univariate and multivariate analysis of progress-free survival in patients with endometrial stromal sarcoma.

**Variables**	**Univariate analysis**		**Multivariate analysis**	
	**HR (95%CI)**	***P*** **-value**	**HR (95%CI)**	***P*** **-value**
Age (years) (>45 or ≤ 45)	1.708 (0.657–4.438)	0.272		
BMI (kg/m^2^) (≥28 or <28)	1.235 (0.281–5.441)	0.780		
Menopausal status (yes or no)	3.254 (1.188–8.914)	0.022		
Tumor size (>7cm or ≤ 7cm)	1.570 (0.523–4.709)	0.421		
Lymphadenectomy (yes or no)	0.749 (0.244–2.298)	0.614		
Ovarian preservation (yes or no)	6.805 (2.416–19.167)	0.000	4.709 (1.071–20.698)	0.040
Histological type				
LG-ESS	1.000	0.001		
HG-ESS	6.246 (2.018–19.336)	0.001		
UUS	10.380 (2.463–43.757)	0.001		
FIGO Stage (I + II or III + IV)	3.210 (1.163–8.860)	0.024		
Adjuvant therapy (yes or no)	1.379 (0.449–4.234)	0.574		
CD10 (positive or negative)	23.210 (0.007–73864.471)	0.445		
ER (positive or negative)	0.142 (0.041–0.488)	0.002		
PR (positive or negative)	0.127 (0.037–0.432)	0.001		
Ki-67 index (≥35% or <35%)	10.702 (3.509–32.633)	0.000	44.374 (4.819–408.617)	0.001

*HR, hazard ratio (HR); CI, confidence interval*.

## Discussion

Our study explored the prognostic value of the Ki-67 index in patients with endometrial stromal sarcoma. We found that the optimal cut-off value of the Ki-67 index for predicting recurrent endometrial stromal sarcoma was 35%. And the Ki-67 index was an important prognostic factor of endometrial stromal sarcoma. The high Ki-67 index (≥35%) had statistically significant shortened progress-free survival and overall survival.

Endometrial stromal sarcoma is a rare gynecological tumor, with a relatively good prognosis. But it is prone to recurrence. The 5-year overall survival of endometrial stromal sarcoma is reported to be 80–100% (20). However, even in the early stages of the patients with low-grade endometrial stromal sarcoma, about one third of the patients have recurrent disease. Since women diagnosed with low-grade endometrial stromal sarcoma are relatively young, it is very important to accurately predict recurrent disease. Because “high-risk” endometrial stromal sarcoma may be considered to require more frequent follow-up or appropriate adjuvant therapy to reduce the risk of recurrence and avoid insufficient treatment, it is necessary to identify high-risk endometrial stromal sarcomas. However, the predictive and prognostic value of immunohistochemical markers in endometrial stromal sarcoma has not been clearly established yet.

Ki-67 is a proliferation marker protein, located in Chromosome 10. Its expression is specific in the cell cycle and can effectively reflect the state of cell proliferation, so it is often used to evaluate the tumor proliferation activity and biological behavior. The Ki-67 index is related to tumor prognosis. Park et al. discussed the potential therapeutic targets and prognostic indicators of the patients with low-grade endometrial stromal sarcoma (5). Their results showed that the expression of the Ki-67 index was significantly correlated with disease-free survival (*P* = 0.005) and overall survival (*P* = 0.018). Our results showed that the high Ki-67 index were significantly associated with poorer progress-free survival and overall survival. The results of multivariate analysis showed that the Ki-67 index was independent prognostic factors of endometrial stromal sarcoma, consistent with literature (5). It has been reported that Ki-67, a proliferation biomarker, can significantly predict recurrent disease. With the increase of the Ki-67 index, the recurrence rate increased significantly in low-grade endometrial stromal sarcoma (21, 22). In our study, the high Ki-67 index was significantly related to recurrence of endometrial stromal sarcoma. For the patients with the high Ki-67 index, more frequent follow-up or appropriate adjuvant treatment can be considered to reduce the risk of recurrence. Furthermore, we initially explored the optimal cut-off value of the Ki-67 index for predicting recurrent endometrial stromal sarcoma. The Ki-67 index has potential value in predicting the recurrence of endometrial stromal sarcoma, but it needs to be further verified by more research.

Our study also showed that the Ki-67 index may be associated with some clinicopathological parameters, such as BMI, menopausal status, histological type, estrogen receptor, and progesterone receptor. In multivariate analysis, we found that not only the Ki-67 index is an independent prognostic factor of endometrial stromal sarcoma, but also ovarian preservation. There is still controversy about the ovarian preservation in endometrial stromal sarcoma. Whether young patients can choose ovary preservation and whether ovary preservation has an effect on survival prognosis of endometrial stromal sarcoma remains to be further explored. Endometrial stromal sarcoma is a hormone-sensitive tumor; ovarian preservation may stimulate tumor growth. Ovarian preservation has been previously reported to be associated with a higher recurrence rate and shorter recurrence free survival in endometrial stromal sarcoma (23, 24). Our results showed that ovarian preservation was bound up with poorer progress-free survival (*P* = 0.000), but not with overall survival (*P* = 0.181). However, our patients were a group of heterogeneous, mixed-stage patients with a limited sample size, and we need to expand the sample size to determine the impact of ovarian preservation on survival and recurrence. Yoon et al. analyzed the prognosis of 114 patients with endometrial stromal sarcoma; their results showed that ovarian preservation was an independent prognostic factor in poor recurrence-free survival, but had no effect on overall survival, which was consistent with our results (25). However, some studies suggest that ovarian preservation has little effect on the prognosis. Shah et al. showed that the 5-year survival rate of the patients with low-grade endometrial stromal sarcoma receiving bilateral salpingo-ovariectomy was not statistically significant compared with those patients who did not receive bilateral salpingo-ovariectomy (92 vs. 94%, *P* = 0.267), especially in Stage I patients (26). In addition, there was no difference in the survival rate between the patients high-grade endometrial stromal sarcoma who received bilateral salpingo-ovariectomy and those who did not (*P* = 0.279). Zhou et al. retrospectively analyzed 114 patients with low-grade endometrial stromal sarcoma, and the results showed that ovarian preservation was not a factor affecting prognosis (27). These findings suggest that the potential benefits of ovarian preservation should be carefully weighed against the increased risk of recurrence.

Although adjuvant therapy and the expression of the estrogen receptor or the progesterone receptor were not significantly associated with prognosis in multivariate analysis, their prognostic value in endometrial stromal sarcoma cannot be denied. It is generally believed that post-operative adjuvant radiotherapy can reduce pelvic recurrence and improve the survival rate of the patients. Weitmann et al. showed that surgery combined with post-operative radiotherapy is an effective treatment for endometrial stromal sarcoma. (28) Post-operative adjuvant chemotherapy has been reported to be associated with an improved disease-free survival rate in patients with high-grade endometrial stromal sarcoma and undifferentiated uterine sarcoma (29). However, our study did not show a clear survival benefit of adjuvant therapy, which may be affected by the rarity and heterogeneity of this disease. Compared with other uterine sarcomas, positive expression rates of the estrogen receptor and the progesterone receptor are higher in endometrial stromal sarcoma, especially in low-grade endometrial stromal sarcoma. It has been reported that the expression of the estrogen receptor and the progesterone receptor in endometrial stromal sarcoma is positively correlated with disease-free survival and overall survival (30, 31). In univariate analysis, our findings support these results. Hormone therapy is an effective treatment option for patients with advanced or recurrent endometrial stromal sarcoma, including progesterone, Gonadotropin-Releasing Hormone agonist, aromatase inhibitors, and so on. A recent meta-analysis of hormone therapy in 315 patients with low-grade endometrial stromal sarcoma has shown that hormone therapy reduced the risk of recurrence in patients in FIGO Stages I-II (32). Immunohistochemical staining to determine the estrogen receptor and the progesterone receptor expression may contribute to the prognosis of the patients with endometrial stromal sarcoma.

There are some limitations in our study. First of all, this study is a retrospective study in a single institution, and multi-center studies are needed in the future. Secondly, the sample size of endometrial stromal sarcoma is small due to the low clinical incidence. Prospective studies that ensure a high number of patients can further confirm the predictive and prognostic value of the Ki-67 index. Furthermore, the predictive value of other immunohistochemical parameters, such as the estrogen receptor and the progesterone receptor, needs to be further explored.

## Conclusions

The Ki-67 index is an immunohistochemical parameter with important clinical value. A Ki-67 index cut-off of 35% was optimal for predicting recurrent endometrial stromal sarcoma. The Ki-67 index may be a useful prognostic marker in endometrial stromal sarcoma.

## Data Availability Statement

The raw data supporting the conclusions of this article will be made available by the authors, without undue reservation.

## Ethics Statement

The studies involving human participants were reviewed and approved by the Ethics Committee of the First Affiliated Hospital of Chongqing Medical University. The patients/participants provided their written informed consent to participate in this study.

## Author Contributions

YM, QQ, and XM: study design. YM, QQ, and YL: data collection. YM, QQ, YL, SR, and FZ: manuscript preparation. YM, SR, and FZ: data analysis and interpretation. YM and XM: manuscript writing. All authors confirm they contributed to manuscript reviews, given final approval of this version to be published, and are responsible for the manuscript content.

## Conflict of Interest

The authors declare that the research was conducted in the absence of any commercial or financial relationships that could be construed as a potential conflict of interest.

## Publisher's Note

All claims expressed in this article are solely those of the authors and do not necessarily represent those of their affiliated organizations, or those of the publisher, the editors and the reviewers. Any product that may be evaluated in this article, or claim that may be made by its manufacturer, is not guaranteed or endorsed by the publisher.
